# Unveiling the Angiogenic Potential and Functional Decline of Valve Interstitial Cells During Calcific Aortic Valve Stenosis Progression

**DOI:** 10.1111/jcmm.70511

**Published:** 2025-03-30

**Authors:** Adeline Blandinières, Elisa Rossi, Nicolas Gendron, Jeanne Rancic, Mickael Rosa, Annabelle Dupont, Salim Idelcadi, Aurélien Philippe, Bastien Poitier, Ivan Bièche, Sophie Vacher, Bernard Cholley, Pascale Gaussem, Sophie Susen, David M. Smadja

**Affiliations:** ^1^ Université Paris Cité Innovative Therapies in Haemostasis, INSERM Paris France; ^2^ AP‐HP, European Georges Pompidou Hospital Hematology Department Paris France; ^3^ Univ. Lille, Inserm, CHU Lille, Institut Pasteur de Lille, U1011‐EGID Lille France; ^4^ AP‐HP, European Georges Pompidou Hospital Cardiac Surgery Department Paris France; ^5^ Université Paris Cité and Pharmacogenomics Unit, Department of Genetics Paris France; ^6^ AP‐HP, European Georges Pompidou Hospital Department of Anesthesia and Intensive Care Paris France

**Keywords:** aortic valve stenosis, endothelial progenitor cells, neovascularisation, perivascular cells, valve interstitial cell

## Abstract

Valve interstitial cells (VICs) play a critical role in aortic valve calcification and angiogenic processes associated with calcific aortic valve stenosis (CAVS). Within the same valve, VICs from differently calcified regions can exhibit diverse phenotypic and functional properties. We hypothesised that VICs isolated from noncalcified (NC‐VICs) and calcified (C‐VICs) areas of human aortic valves possess distinct angiogenic characteristics. In this study, we isolated C‐VICs and NC‐VICs from 23 valves obtained after aortic valve replacement due to CAVS. Both VIC types exhibited similar phenotypes in culture, characterised by morphology, expression of mesenchymal/fibroblastic markers, proliferation and osteogenic differentiation. No significant differences were observed in the secretion of angiogenic factors, including VEGF‐A, Ang‐1, Ang‐2, PlGF, bFGF between NC‐VICs and C‐VICs. However, when co‐injected with endothelial colony‐forming cells (ECFCs) into Matrigel implants in vivo in mice, implants containing NC‐VICs showed significantly higher microvessel density compared to those with C‐VICs (*p* < 0.001). Additionally, NC‐VICs co‐cultured with ECFCs expressed significantly higher levels of the perivascular markers αSMA and calponin compared to C‐VICs (*p* < 0.001 and *p* < 0.05, respectively). In conclusion, our study reveals the heterogeneity in VIC plasticity within the aortic valve during CAVS. The diminished capacity of VICs from calcified areas to differentiate into perivascular cells suggests a loss of function as valve disease progresses. Furthermore, the ability of VICs to undergo perivascular differentiation may provide insights into valve homeostasis, angiogenesis and the exacerbation of calcification.

## 1 Introduction

Calcific aortic valve stenosis (CAVS) is a mechanism of progressive fibrocalcific remodelling of the aortic valve that leads to the narrowing of the aortic valve orifice and obstruction of blood flow [1, 2]. In 2017, the prevalence of CAVS was estimated to be 12.6 million cases per year worldwide and resulted in 102,700 deaths per year [3]. To date, there is no preventive or curative pharmacological treatment, and it is necessary to resort, when possible, to an invasive aortic valve replacement [1]. CAVS pathogenesis remains elusive but associates active calcific lesion formation with extracellular matrix (ECM) remodelling [1] and accumulation of inflammatory cells and lipids [2, 4]. While healthy valves are usually completely avascular, a process of neovascularisation within the pathological valve has been described [5, 6, 7, 8] and may promote calcification by providing essential supplies to pathologically thickened tissue.

Neovascularisation within valves has been associated with an imbalance between pro‐angiogenic and anti‐angiogenic factors [4, 5, 8, 9, 10], but valve interstitial cells (VICs) may also play a role in these neovascularisation processes [10]. VICs are the predominant cells in the aortic valve; they can be found in the three layers of the valve: fibrosa, spongiosa and ventricularis. VICs are responsible for ECM regulation and to ensure tissue homeostasis. When co‐cultured with valve endothelial cells (VECs), porcine VICs adopt a pericyte‐like behaviour and stabilise early VECs vasculogenic network formation [11]. We recently demonstrated that human VICs isolated from the noncalcified part of CAVS valves were angiogenic cells in vivo and in vitro by secreting VEGF‐A and differentiating into perivascular cells when co‐cultured with endothelial colony‐forming cells (ECFCs) which are cells known to display vasculogenic capacities [9].

This initial work was conducted only with VICs isolated from noncalcified areas, whereas phenotypic and functional heterogeneity among these cells has been highlighted. In healthy aortic murine valves, several populations of VICs that present distinct functional properties can be identified within the valve leaflets with a precise distribution in space [12]. In human aortic valve, fibrosa‐derived VICs showed a higher calcification potential than ventricularis‐derived VICs associated with a specific protein signature [13]. Furthermore, in calcified valves, distinct stages of disease progression can be identified within the same leaflet that have specific transcript and protein expression [13] suggesting that cells isolated from these different areas may have different properties.

Given the proposed spatial heterogeneity of VIC populations across the valve leaflets, the objective of this study was to compare the angiogenic potential of human VICs isolated from calcified (C‐VICs) and noncalcified (NC‐VICs) regions of the aortic valve.

## 2 Material and Methods

### 2.1 Study Population

Patients were recruited in the cardiovascular surgery department of the Hôpital européen Georges Pompidou (AP‐HP, Paris, France). The study was approved by the local Ethics committee (CELLBIOPEX, registry number 2017‐A03554‐4). Informed consent was obtained from each patient included. Inclusion criteria comprised the indication of aortic valve replacement for severe CAVS according to American Heart Association guidelines [14, 15] excluding other causes of valve replacement (i.e., endocarditis). When echocardiography data were available, most patients exhibited at least one criterion for severe CAVS (aortic valve area < 1.0 cm^2^, peak velocity > 4 m/s or mean gradient > 40 mmHg) [16, 17]. One single patient has been classified as severe even if gradient and Vmax were low. Indeed, as described in recent recommendations [15] if the patient has low flow (low stroke volume or reduced left ventricular ejection fraction) he can be classified as severe. Aortic valve was recovered after aortic valve replacement for severe CAVS. Bicuspid valves were excluded from the study. Demographic, comorbidities, clinical and functional data were prospectively recorded. Human aortic valves were obtained immediately after surgery and kept in NaCl 0.09% at 4°C for up to 24 h before processing.

### 2.2 Aortic VIC Isolation and Culture

**FIGURE 1:**
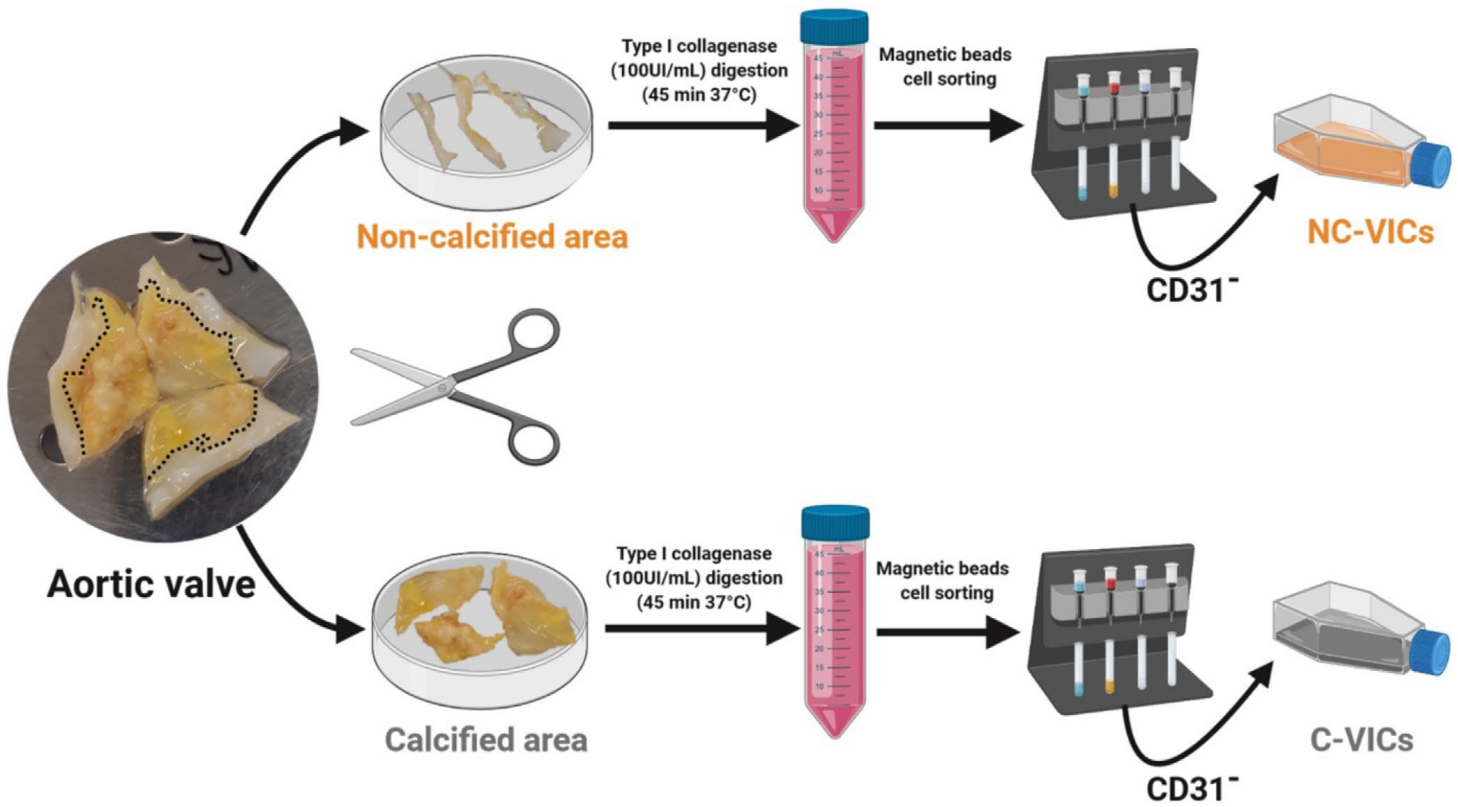
Isolation process of VICs from calcified (C‐VICs) and noncalcified (NC‐VICs) area of the aortic valve. Noncalcified and calcified areas shown are representative of the complete cohort.

Calcified and noncalcified areas of the valve were separated according to macroscopic appearance and processed separately (Figure 1). Calcified and noncalcified areas of each of the three leaflets of the valve were mixed together. C‐VICs and NC‐VICs were obtained after type‐I collagenase digestion (Life Technologies, USA) of the aortic valve, as previously described [9, 18]. After digestion, single‐cell suspension was depleted of CD31‐positive cells with anti‐CD31‐coated magnetic beads (Dynabeads CD31 Endothelial Cell, Invitrogen, USA) according to the manufacturer's instructions. NC‐VICs and C‐VICs from the CD31‐negative cell suspension were seeded on a noncoated surface and cultured in Smooth Muscle Growth Medium‐2 (SMGM2, Lonza, USA). Absence of contamination by endothelial cells and leucocytes was ensured after digestion by flow cytometry, as previously described [9].

### 2.3 ECFCs and Mesenchymal Stromal Cell Isolation and Culture

Cord blood ECFCs were isolated from cord blood adherent mononuclear cell (MNC) fraction as previously described and according to current recommendations [19, 20, 21]. Human umbilical cord blood samples from healthy full‐term newborns were obtained from the Cell therapy Unit of Saint‐Louis Hospital (AP‐HP, Paris, France) which is authorised by the French Regulatory Authority (authorisation no. PPC51). ECFCs were expanded on fibronectin (FN)‐coated plates (Merck, Germany) using EGM‐2MV (Lonza, USA) supplemented with 10% heat‐inactivated foetal bovine serum (FBS) and used between passages 3 and 6. Mesenchymal stem cells (MSCs) were isolated from the MNCs fraction of healthy donor human adult bone marrow also obtained from the Cell therapy Unit of Saint‐Louis Hospital (AP‐HP, Paris, France, authorisation no. PPC51), as previously described [22] and cultivated in α‐MEM medium containing GlutaMAX (Gibco, USA) supplemented with 10% FBS and 1 ng/mL basic fibroblast growth factor (bFGF, R&D Systems, USA). MSCs were used as a positive control to validate the mesenchymal phenotype of VICs, ensuring that their behaviour could be contrasted against a well‐established mesenchymal cell model.

### 2.4 Flow Cytometry Immunophenotyping

Cultured cells were detached with trypsin (Gibco, USA), washed in phosphate‐buffered saline (PBS) containing 10% FBS and resuspended in PBS/0.5% BSA (Bovine serum albumin, Sigma Aldrich, USA) at a concentration of 10^6^ cells/100 μL. Cells were incubated with PE conjugated anti‐CD90 (dil 1/10, IM1840, Beckman Coulter, USA) and APC‐Cy7‐conjugated anti‐CD31 (dil 1/30, WM59, Biolegend, USA) for 30 min at 4°C away from light. Isotype‐matched antibodies from the same manufacturer were used as a negative control (A07796, Beckman Coulter, USA and MOPC‐21, Biolegend, USA). Acquisition of 20,000 events was performed on the Attune acoustic flow Cytometer (Life Technologies, USA) and analysed on Attune cytometer software (Life Technologies, USA).

### 2.5 Real‐Time Reverse Transcription Polymerase Chain Reaction

RNA was extracted using RNeasy Micro Kit (Qiagen, Germany) according to the manufacturer's instructions. cDNA synthesis was performed using the QuantiTect Reverse Transcription kit (Qiagen, Germany). For quantification of COLA1, FN1, THY1, CD44, CDH2, S100A4, TWIST1, VIM, NT5E, EBTPD1, ADORA2B, P2RY2, P2RY12 and S1PR2, polymerase chain reaction was performed as previously described [9]. Primer sequences are available in Table S1. Transcripts of TBP gene, encoding the TATA box‐binding protein, were used as endogenous control. Results are expressed as ‘normalised mRNA level’ as previously described [23]. Results, expressed as *N*‐fold differences in target gene expression relative to the TBP gene, and termed Ntarget, were determined with the formula: Ntarget = $2^{\Delta C_{t}\;\text{Sample}}$, where the Δ*C*_t_ value of the sample was determined by subtracting the *C*_t_ value of the target gene from the *C*_t_ value of the TBP gene. The Ntarget values of the samples were subsequently normalised, that is Ntarget value was divided by the Ntarget value of the smallest quantifiable amount of target gene mRNA (ie, target gene *C*_t_ value = 35).

### 2.6 VIC Osteogenic Cell Differentiation

Cells at 80% confluence were incubated in six‐well microplates in osteogenic medium consisting of Dulbecco's modified Eagle's medium (DMEM, Invitrogen, USA) with 4.5 g/L glucose, 10% FBS, 10^−7^ M dexamethasone, 50 μg/mL ascorbic acid, 100 U/mL penicillin, 0.1 mg/mL streptomycin and 3 mM inorganic phosphate as previously described [9, 23]. The medium was replaced every 3 days for 14 days. To assess calcium accumulation, cells were fixed in 4% paraformaldehyde (PFA) and stained for 10 min with 1 mL of 40 mM Alizarin Red (Sigma, France). Quantification of Alizarin Red staining was performed after dilution in 10% (v/v) acetic acid by reading the optical density (OD) by spectrophotometry at 405 nm wavelength (Fluostar optima, BMG Labtech, Germany) as previously described [9, 24].

### 2.7 Adipogenic Cell Differentiation

Cells at passage 4–5 at 80% confluence were incubated in six‐well microplates in adipogenic medium consisting of Dulbecco's modified Eagle's medium (DMEM, Invitrogen) with 1 g/L glucose, 10% FBS, 0.5 mM isobutylmethylxanthine (Sigma, France), 60 μM indomethacine (Sigma, France), 10^−6^ M dexamethasone, 100 U/mL penicillin and 0.1 mg/mL streptomycin as previously described [23]. The medium was replaced every 3 days for 21 days. Cells containing lipid vacuoles were observed by phase‐contrast microscopy.

### 2.8 VIC Population Doubling

For population doubling studies, cells were serially passaged at a density of 5000 cells per cm^2^ as soon as they reached confluence. Population doubling was determined using the following formula: log_2_(nf/n0), where n0 was the cell number initially seeded and nf the cell number at confluence obtained at each passage as previously described [25]. Cumulative population doubling was the sum of all previous population doublings.

### 2.9 Soluble Factors Secreted by C‐VICs and NC‐VICs

Cells in culture were grown for 48 h in SMGM‐2 supplemented with 0.5% FBS. Conditioned medium (CM) was harvested, centrifuged twice (at 405 *g* for 5 min then at 16435 *g* for 2 min) and stored at −80°C. Basic fibroblast growth factor (bFGF), placental growth factor (PlGF), soluble E‐selectin, angiopoietin (Ang)‐1 and Ang‐2 concentrations were quantified in VICs‐CM with a Human Magnetic Luminex Assay (R&D systems, France). Data were assessed with the Bio‐Plex 200 using the Bio‐Plex Manager 5.0 software (Bio‐Rad, France) (*n* = 3 for each condition). The concentration of VEGF‐A was quantified by ELISA (R&D systems, France) (*n* = 8 for each condition).

### 2.10 In Vivo Matrigel Implant Assay

All animal experiments were approved by the ethic committee Paris Descartes (CEEA 34), project n°2019071212082084. For Matrigel implant assay, 1.5 × 10^6^ ECFCs and 1.5 × 10^6^ MSCs, 1.5 × 10^6^ NC‐VICs or 1.5 × 10^6^ C‐VICs were resuspended in 200 μL of Matrigel (BD MatrigelMatrix, BD Biosciences, USA) and injected subcutaneously in the back of NMRI‐Fox1^nu^/1^nu^ MALE mice (Janvier Laboratories, France). Manipulation of Matrigel before injection was done on ice to prevent early polymerisation. Implants were removed after 10 days under anaesthesia by a mix of oxygen–isoflurane 2%. Implants were fixed in 4% PFA and embedded in paraffin for histologic analysis. Sections (4 μm) from Matrigel plugs were processed by deparaffinisation (Xylene, EtOH 100%, EtOH 95%, EtOH 70%, PBS 1× each 10 min/twice). Haematoxylin and eosin staining was performed, and blood vessel infiltration was quantified by Image J as previously described [26].

### 2.11 Immunofluorescence Studies

Culture chamber slides were coated with FN and seeded with ECFCs + NC‐VICs or with ECFCs + C‐VICs at a 1:1 ratio. After co‐culture for 7 days, VICs differentiate into perivascular cells [9]. Once differentiated, the cells were fixed with cold pure methanol on ice for 10 min. For ECFCs immunostaining, samples were incubated with a mouse antibody against human von Willebrand factor (1:200, M 0616, Dako, Denmark) for 1 h at room temperature (RT), followed by the secondary antibody Texas Red anti‐mouse IgG (1:500, TI‐2000‐1,5, Vector, USA) for 1 h at RT. Differentiated NC‐VICs or C‐VICs were incubated with anti‐human calponin (1:100, ab46794, Abcam, UK), and anti‐human alpha smooth muscle actin (αSMA, 1:200, F3777, Sigma, France) or a negative control antibody (Sc‐3878, Santa Cruz, USA). After washing, samples were incubated with the appropriate FITC‐labelled secondary antibody, when necessary (1:500, FI‐1000 or FI‐2000‐1.5, Vector, USA), and nuclei were counterstained and mounted using Vectashield with DAPI (H‐1200‐10, Vector, USA). Green fluorescence and nuclei number were quantified by Image J.

Regarding histological sections from mouse plugs, antigenic unmasking solution immune Retriever 20× diluted 1× pH 9 with EDTA and a blocking passage in PBS 1×, goat serum 10% + Triton 1% – 30 min at RT were performed. Incubation with primary antibodies: negative controls Mouse (Sc‐3878, IgG2a), anti‐αSMA (1: 100, F3777, Sigma, France) and human anti‐CD31 (1: 200, 555,444, BD Pharmigen, USA), was incubated overnight and followed by the appropriate secondary antibodies (Alexa 488, A10680, Invitrogen, USA and Alexa 555, A21422, Invitrogen, USA). After washing with PBS 1X, slides were counterstained and mounted by DAPI‐VectaShield (H‐1200‐10, Vector, USA). Fluorescence images were taken using the Leica TCS SP8 X confocal system. Blood vessel infiltration was quantified by ImageJ.

### 2.12 Statistical Analysis

Data are shown as mean ± standard error of the mean (SEM) or median (interquartile range). Data sets failed normality and equal variance tests were analysed using the Mann–Whitney nonparametric test or Kruskal–Wallis test followed by the Dunn multiple comparisons test. All statistical analyses were performed using GraphPad Prism 5 software (GraphPad Software Inc., San Diego, CA). Differences were considered significant at *p* < 0.05 and shown as *p < 0.05, **p < 0.01 and ***p < 0.001.

## 3 Results

### 3.1 VICs Isolated From Calcified and Noncalcified Aortic Valve Area Exhibit a Similar Phenotype in Culture

**FIGURE 2:**
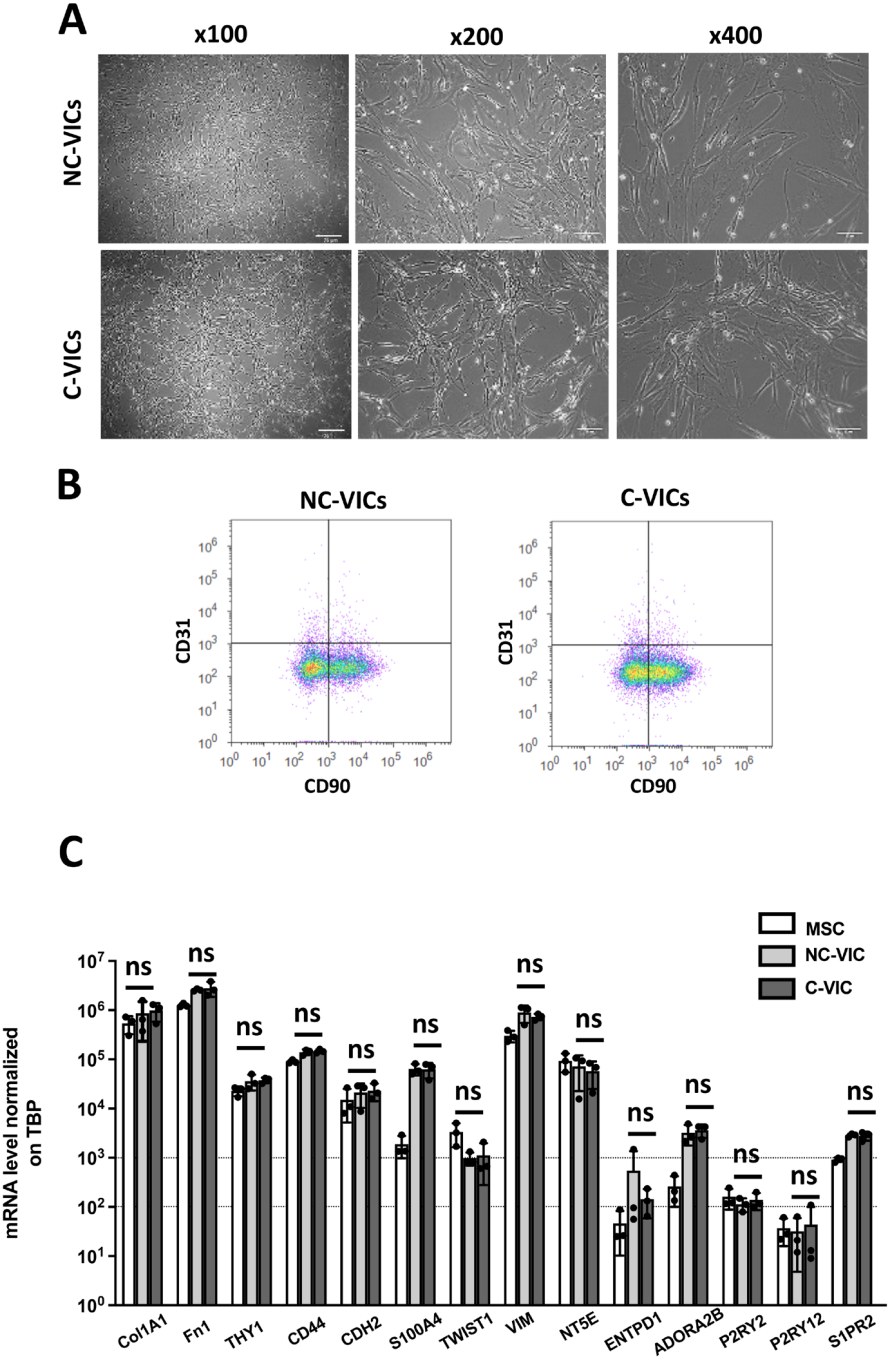
Characterisation of NC‐VICs and C‐VICs. (A) Morphology. Phase‐contrast image of VICs isolated from NC‐VICs and C‐VICs in culture at different magnifications (×100, ×200, ×400). (B) CD90 and CD31 expression in acoustic cytometry by NC‐VICs and C‐VICs (*n* = 6) (C) Col1A1, Fn1, THY 1, CD44, CDH2, S100A4, TWIST1, VIM, NT5E, ENTPD1, ADORA2B, P2RY2, P2RY12, S1PR2 mRNA expression by MSCs, C‐VICs and NC‐VICs (*n* = 3, mean ± SEM). ns, *p* > 0.05.

**FIGURE 3:**
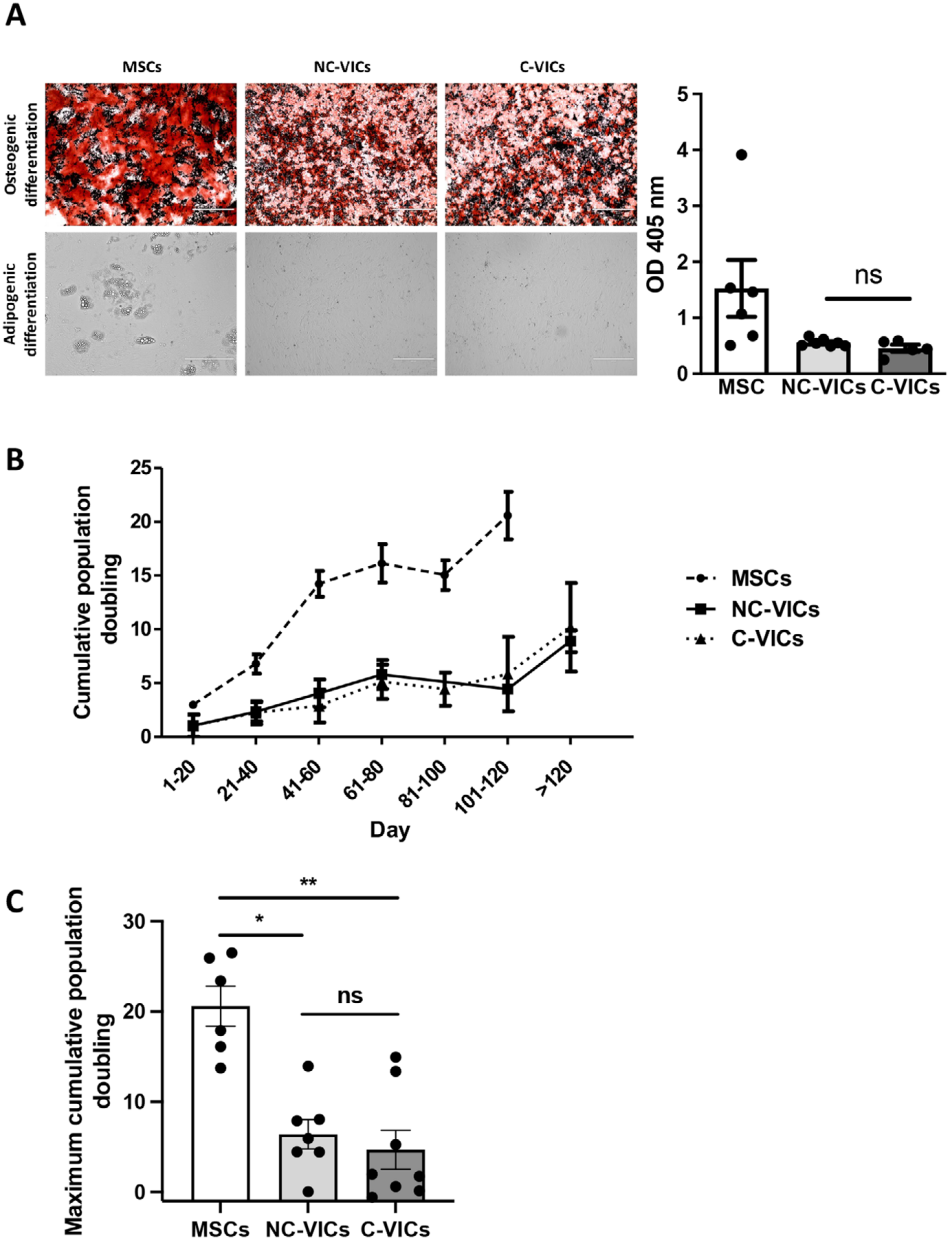
Functional characterisation of NC‐VICs and VICs. (A) Osteogenic differentiation of MSC, VICs isolated from NC‐VICs and C‐VICs revealed by Alizarin Red staining. Adipogenic differentiation of MSCs revealed by the presence of lipid drops on phase‐contrast image (scale 400 μm). Quantification of osteogenic differentiation of MSCs, NC‐VICs and C‐VICs (OD 405 nm, *n* = 5 to 7) (B) Cumulative population doubling of MSC, NC‐VICs and C‐VICs (*n* = 4). *C.* Maximum cumulative population doubling of MSC, NC‐VICs and C‐VICs (*n* = 4). ns, *p* > 0.05, *p < 0.05 and, **p < 0.01.

Between February 2019 and January 2020, a total of 23 patients, including 16 men and 7 women with a median age of 71 years, were included in the study (described in Table 1). After a few days in culture, human C‐VICs and NC‐VICs displayed similar morphology: the elongated, spindle‐shaped aspect characteristic of mesenchymal/fibroblastic cells in culture (Figure 2A). Both C‐VICs and NC‐VICs expressed the mesenchymal marker CD90 (THY‐1) at variable levels (% of C‐VICs CD90^+^: median = 53.4% [44.6–84.4] and % of NC‐VICs CD90^+^: median = 58% [46.2–86.55]) and were negative for the endothelial marker CD31 (PECAM‐1) in cytometry (Figure 2B). Moreover, expression of mesenchymal and fibroblastic genes (COL1A1, FN1, THY‐1, CD44, CDH2, S100A4, TWIST1, VIM) or sphingosine‐1‐phosphate receptor and purinergic signalling mediators (ADORA2B, P2RY2, P2RY12, S1PR2, 5′‐Nucleotidase Ecto: NT5E/CD73 and Ectonucleoside Triphosphate Diphosphohydrolase 1: ENTPD1/CD39) was quantified by real‐time quantitative polymerase chain reaction. NC‐VICs and C‐VICs expressed all above‐mentioned genes with no significant difference between both types of VICs (*p* > 0.05 for each, Figure 2C). VICs are known to have pluripotent differentiation capacities [27]. When cultivated in an osteogenic medium, both C‐VICs and NC‐VICs differentiated towards the osteogenic lineage (Figure 3A). Quantification of calcium deposits after Alizarin Red staining showed no difference between both types of VICs (*p* > 0.05, Figure 3A). When both types of VICs were cultivated in adipogenic medium, no formation of lipid vacuoles, typical of adipogenic differentiation, was observed as previously described [9]. In contrast, lipid vacuoles were observed in MSCs, which are known to have adipogenic differentiation properties [28] (Figure 3A). C‐VICs and NC‐VICs demonstrated a similar population doubling time; however, a lower maximum cumulative population doubling was shown in contrast to MSCs (*p* < 0.05, Figure 3B,C).

**TABLE 1:** Characteristics of the patients: Demographic data, risk factors of aortic valve stenosis, preoperative cardiovascular status, functional symptoms and echocardiography data.

Number of patients	*n* = 23
**Demographic data**	
Age (year) – median [min–max]	71 [46–80]
Sex ratio (M/F)	16/7
**Risk factors**	
BMI (Kg/m^2^) – median [min–max]	27.6 [18.8–37.6]
Active smoking – y/n (%)	8/15 (23%)
Dyslipidemia – y/n (%)	15/8 (65%)
Type 1 diabetes – y/n (%)	0/23 (0%)
Type 2 diabetes – y/n (%)	6/17 (26%)
High blood pressure – y/n (%)	16/7 (70%)
Chronic kidney failure – y/n (%)	4/19 (17%)
**Co‐medication**	
Statins – y/n (%)	12/11 (52%)
Beta‐blockers – y/n (%)	10/13 (43%)
ACE inhibitor/ARBs – y/n (%)	12/11 (12%)
Calcic inhibitors – y/n (%)	9/14 (39%)
Loop diuretics – y/n (%)	6/17 (35%)
Thiazide diuretics – y/n (%)	6/17 (35%)
Anticoagulants – y/n (%)	6/17 (26%)
Antiplatelet drugs – y/n (%)	10/13 (12%)
**Preoperative cardiovascular status**	
Critical leg ischemia – y/n (%)	1/19 (17%)
Myocardial infarction – y/n (%)	1/22 (4%)
Stable angina – y/n (%)	4/19 (17%)
**Functional symptoms**	
Dyspnea – y/n (%)	17/6 (74%)
Syncope – y/n (%)	2/21 (9%)
Exertional angina – y/n (%)	4/19 (17%)
NYHA score	
1 (*n*)	2
2 (*n*)	7
3 (*n*)	8
4 (*n*)	0
No information (*n*)	6
**Echocardiography**	
Peak velocity (m/s) – median [min–max]	4.55 [2.34–5,82]
No information (*n*)	5
Peak velocity (m/s) < 4 (*n*)	4
Peak velocity (m/s) > 4 (*n*)	14
Mean gradient (mmHg) – median [min–max]	54 [13–90]
No information (*n*)	4
Mean gradient (mmHg) < 40 (*n*)	6
Mean gradient (mmHg) > 40 (*n*)	13
Aortic valve area (cm^2^) – median [min–max]	0.70 [0.45–0.90]
No information (*n*)	12
0.75 < Aortic valve area (cm^2^) < 1 (*n*)	5
Aortic valve area (cm^2^) < 0.75 (*n*)	6
Left ventricular ejection fraction (%)‐ median [min–max]	60 [35–71]

Abbreviations: ACE, angiotensin‐converting enzyme; ARBs, angiotensin receptor blockers; y/n, yes/no.

### 3.2 NC‐VICs Have Increased Perivascular Differentiation Capacity Compared to C‐VICs

**FIGURE 4:**
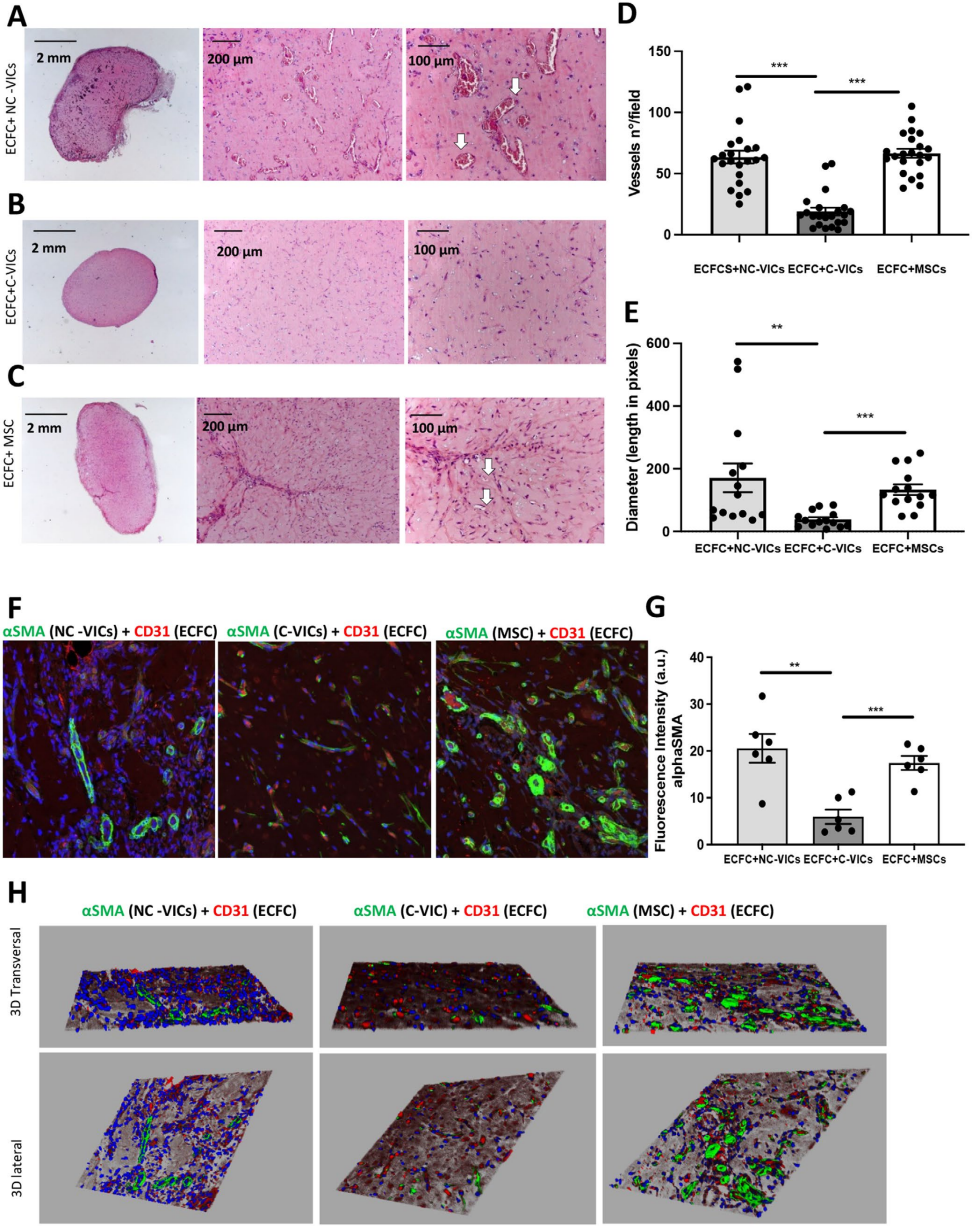
Blood vessels infiltration evaluated by haematoxylin and eosin (H&E) staining. (A) Matrigel plug of ECFCs+NC‐VICs at lower (1,25× left pannel) and higher magnification (10× central, 20× right panel) (B) Matrigel plug of ECFCs+C‐VICs at lower and higher magnification (same order) (C) Matrigel plug of ECFCs+MSCs at lower and higher magnification (same order). Arrows indicate functional vessels containing erythrocytes. (D) Vessels number quantification (*n* = 10 matrigel plugs for each group) (E) Diameter quantification of *n* = 10 Matrigel plugs for each group (A–C). **p < 0.01 and ***p < 0.001. (F) Matrigel plug section of ECFCs+NC‐VICs, ECFCs+C‐VICs, ECFCs+MSCs (from left to right). CD31 stain (red) was performed to identify endothelial cells while perivascular cells are stained by αSMA (green). (G) Quantification of αSMA stain in *n* = 10 different Matrigel plugs for the 3 different groups mentioned (H) 3D reconstruction of the confocal images proposed in (F).

**FIGURE 5:**
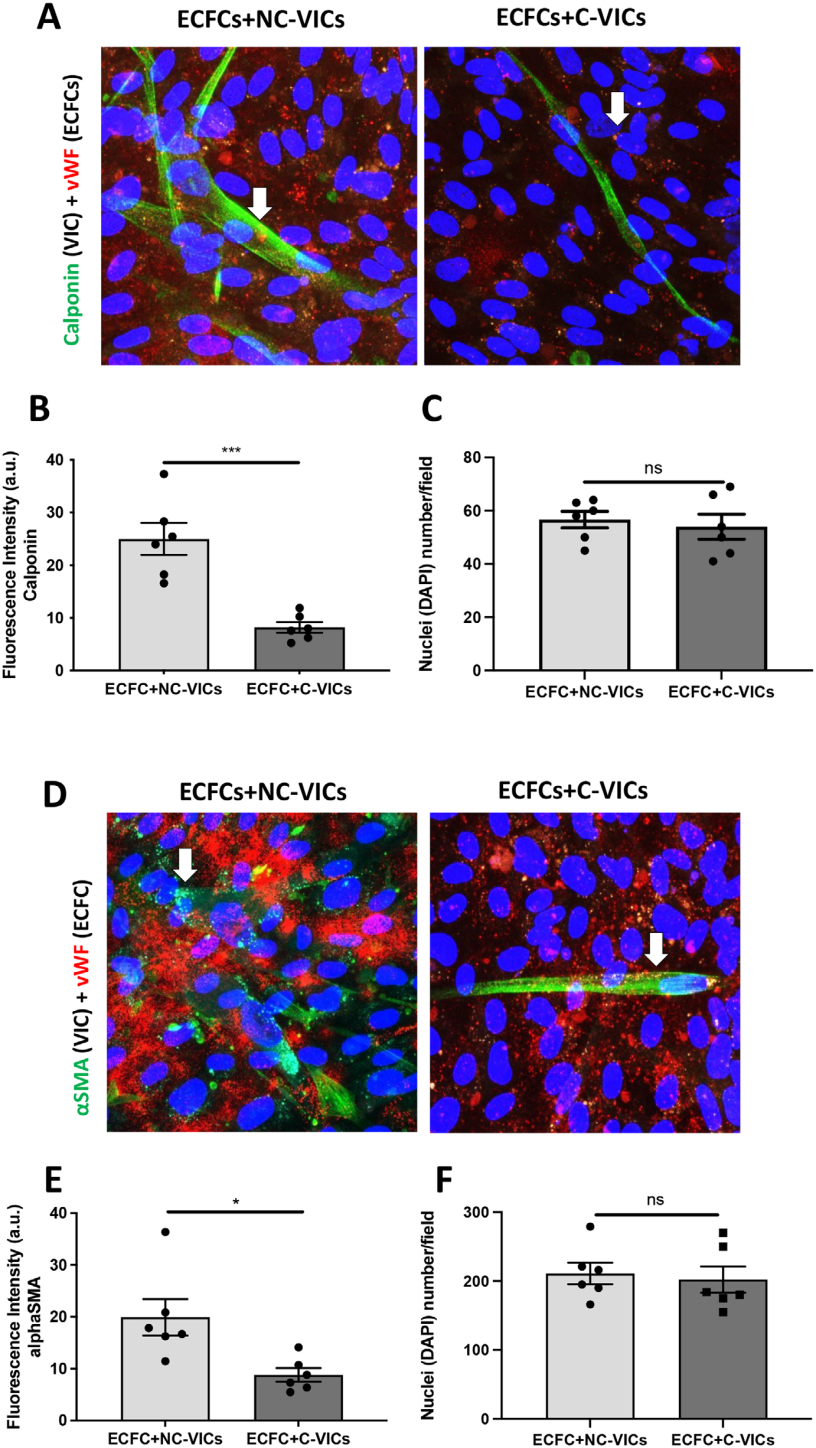
Co‐culture of ECFCs + NC‐VICs, ECFCs + C‐VICs (A) Co‐culture of ECFCs+NC‐VICs and ECFCs+C‐VICs (400× magnification, zoom), ECFCs are positive for von Willebrand factor (vWF) stain (red) and NC‐VICs or C‐VICs are stained for calponin (green) (B) Quantification of calponin stain in *n* = 3 different co‐culture in duplicate (C) Quantification of nuclei in co‐culture of ECFCs+NC‐VICs and ECFCs+C‐VICs stained by calponin (D) Co‐culture of ECFCs+NC‐VICs and ECFCs+C‐VICs (400× magnification, zoom), ECFCs are positive for vWF stain (red) and NC‐VICs or C‐VICs are stained for αSMA (green). (E) Quantification of αSMA stain in *n* = 3 different co‐culture in duplicate (F) Quantification of nuclei in co‐culture of ECFCs+NC‐VICs and ECFCs+C‐VICs stained by αSMA. *p < 0.05 and ***p < 0.001.

To assess whether human aortic VICs isolated from noncalcified and calcified areas were able to modulate angiogenesis in a similar pattern, we first evaluated main angiogenic factors' concentrations in CM from C‐VICs and NC‐VICs: VEGF‐A and all other angiogenic factors quantified (Ang‐1, Ang‐2, PlGF and bFGF) were not significantly different between both cell types (*p* > 0.05 each, Table 2). Moreover, soluble E‐selectin could not be detected in either of the CM result consistent with the negative CD31 sorting performed during VIC isolation. We then evaluated C‐VICs and NC‐VICs perivascular differentiation capacities in a Matrigel implant model in vivo. NC‐VICs and C‐VICs were suspended in Matrigel with ECFCs and injected subcutaneously into nude mice as previously described [9, 26]. Control Matrigel plugs used MSCs associated with ECFCs. After 10 days, explanted Matrigel plug sections containing VICs + ECFCs and MSCs + ECFCs showed numerous vascular channels filled with red blood cells. Interestingly, Matrigel explants containing C‐VICs + ECFCs showed lower microvessel density (18.9 ± 7.2 vessels/field or 417 ± 14 vessels/plug) compared to control explants MSCs + ECFCs (66.6 ± 8 vessels/field or 1465 ± 17 vessels/plug) or NC‐VICs + ECFCs (63.0 vessels/field or 1396 ± 24 vessels/plug) (*p* < 0.001, Figure 4A–D). Interestingly, diameters of vessels formed in Matrigel plugs containing NC‐VICs + ECFCs or MSCs + ECFCs were larger than those formed within Matrigel plugs containing C‐VICs + ECFCs (*p* < 0.001 and *p* < 0.01 respectively, Figure 4E). Immunofluorescence analysis showed perivascular cells positive for αSMA surrounding CD31‐positive blood vessels. In sections of Matrigel plugs containing MSC + ECFCs, αSMA positive cells formed a thick layer around vessels whereas, in sections of Matrigel plugs containing VIC‐derived pericytes, the layer was thinner around large vessels (Figure 4F–H). This discrepancy in perivascular differentiation capacities of C‐VICs and NC‐VICs seen in Matrigel plugs quantification (Figure 4G) was confirmed by a co‐culture experiment in vitro. C‐VICs and NC‐VICs were cultured at a 1:1 ratio with ECFCs. After 7 days of co‐culture, both types of VICs acquired a perivascular phenotype as shown by the induction of expression of calponin and αSMA markers (Figure 5A,D). However, fluorescence intensity was higher in co‐culture with NC‐VICs than C‐VICs (*p* < 0.001 and < 0.05 respectively, Figure 5B,E) confirming a greater capacity of NC‐VICs to differentiate into perivascular cells. To avoid potential interference related to the number of cells, nuclei were numbered in both conditions and no difference was shown (*p* > 0.05 each, Figure 5C,F).

**TABLE 2:** Secretome analysis of noncalcified (NC) and calcified (C) VICs.

Protein	NC‐VICs	C‐VICs	*p*
VEGF‐A	5.98 ± 1.89	7.11 ± 1.43	*p* = 0.78
bFGF	0.099 ± 0.08	0.0442 ± 0.018	*p* = 0.70
PlGF	0.21 ± 0.19	0.00687 ± 0.007	*p* = 0.20
Ang‐1	83.45 ± 70.0	43.45 ± 16.81	*p* = 1.00
Ang‐2	24.26 ± 10.45	41.83 ± 5.78	*p* = 0.40
Soluble E‐selectin	Nd	Nd	

*Note:* Results are expressed in ng/10^6^ cells.

Abbreviation: nd, not determined (under limit of detection).

## 4 Discussion

This study reveals, for the first time, that VICs from calcified areas, compared to noncalcified regions, have a diminished capacity to differentiate into perivascular cells, highlighting their unique angiogenic potential based on aortic valve tissue areas.

Neovascularisation plays a role in the pathogenesis of CAVS and is associated with leaflet remodelling. Although the precise function of neovascularisation remains unclear, it is speculated that it may serve to ensure an adequate oxygen supply to valvular cells in cases where the thickening of the valve hinders sufficient oxygen diffusion directly from the bloodstream. The process of angiogenesis could support the recruitment of inflammatory and osteoprogenitor cells. Given the established correlation between the extent of the inflammatory infiltrate, the degree of neovascularisation and the degree of calcification, this neoangiogenic process holds significance [1, 8]. Interestingly, this process has been described to be localised to the vicinity of calcific nodules [5, 29]. In the moderate form of the disease, angiogenesis has been identified as particularly noteworthy [6]. Collectively, these findings indicate that neovascularisation plays a crucial role in the initial development of calcific nodules but becomes secondary once they are formed. Our results further support this hypothesis. As shown in the model of Matrigel implants, NC‐VICs, derived from regions where the calcification process is ongoing, exhibit the capability for perivascular cell differentiation when associated with ECFCs, similarly with other types of cells such as MSCs [30, 31, 32, 33]. On the contrary, C‐VICs from already calcified areas have lost this ability. Additionally, it is noteworthy that valve neovascularisation is characterised as permeable, potentially leading to intravalve haemorrhage, which could enhance inflammation and contribute to the calcification process [34]. Vessels formed in implants containing VICs, in contrast to MSCs tested as controlled cells here, have a wide lumen but a thinner layer of pericytes. Vessels formed with VIC‐derived pericytes therefore appear to be less robust and may be associated with the haemorrhages observed histologically.

VICs have already been separated according to valve leaflet [13] and undergo significant changes in gene expression and cellular functions during maturation [12]. Indeed, similarly to endothelial and immune cells, VICs demonstrate different populations, in particular concerning ECM production ability during maturation, suggesting specific cell contributions to aortic valve homeostasis and calcific disease mechanisms [12]. In addition, our previous work demonstrated changes in VEGF‐A expression during disease progression, indicating a potential evolution of the angiogenic phenotype as the disease advances [9]. A critical balance between VICs and VECs was also suggested, with each cell type influencing the differentiation of the other [35, 36]. Disruption of this balance, potentially due to impaired VIC‐VEC communication, could drive the pathological progression towards osteogenesis.

The discrepancy in terms of perivascular differentiation ability that we observed between VICs isolated from calcified and noncalcified valve tissue could be a consequence of a dynamic recruitment and/or selection of particular VIC phenotypes throughout the calcification process. In addition, we can also hypothesise that VICs prone to perivascular differentiation are initially more prominent in the valve areas, which will therefore be the most sensitive to calcification during CAVS. This hypothesis is supported by our observation of a regular repartition of calcified regions in the valves studied. To note, the different areas of valve leaflets are subject to different haemodynamic forces that may impact the phenotype of the cells [37]. This hypothesis of specific VICs valve areas is in line with what is observed in human bicuspid aortic valves, which are susceptible to early calcification [38]. In our study, we solely isolated VICs from tricuspid aortic valve. Therefore, to confirm our hypothesis that increased calcification could be linked to modified VIC angiogenic phenotype or production of ECM, further studies should evaluate VICs from calcified and noncalcified areas of bicuspid valve.

This discovery of differential perivascular differentiation of VIC requires further exploration in a relevant in vivo or ex vivo model to fully understand its implications for calcification processes, in particular whether angiogenic properties are a cause or a consequence of calcification. These studies are essential to determine whether targeting the differentiation potential of VICs could represent a viable therapeutic approach. Indeed, VIC differentiation might be associated with disease progression, although this hypothesis remains to be validated. Differentiated VICs could influence the local microenvironment, thereby modulating calcification processes. On the contrary, the effect of a calcifying environment on VIC phenotype also needs to be elucidated. For instance, the loss of perivascular differentiation capacities of C‐VICs could be the result of a feedback loop in which calcified environments suppress VIC plasticity. However, the imbalance between angiogenic and calcification potential of VIC remains a critical issue. Future studies should also focus on molecular characterisation to unravel pathways involved in VIC differentiation and identify potential therapeutic targets. Pharmacological agents or gene‐editing strategies targeting these pathways hold promise for modulating phenotypic changes of VICs.

Our study demonstrates that VICs derived from calcified regions of aortic valves (C‐VICs) exhibit impaired perivascular differentiation compared to those from noncalcified regions (NC‐VICs). Given the established role of oxidative stress in cardiovascular pathologies, it will be imperative to consider in the future whether reactive oxygen species (ROS) and NOX‐mediated mechanisms contribute to VIC dysfunction in the context of calcific aortic valve stenosis (CAVS). Oxidative stress has been implicated in endothelial dysfunction, fibrosis and calcification in various cardiovascular diseases. A previous study by Dushpanova et al. [39] demonstrated that von Willebrand Factor (vWF) expression is linked to NOX‐mediated superoxide production, revealing a novel role of vWF in endothelial dysfunction through its modulation of NADPH oxidase (NOX) activity and endothelin‐1 (ET‐1) expression. This suggests that oxidative stress may also influence VIC plasticity and function in aortic valve pathology. Given that vWF is an important regulator of angiogenesis and haemostasis, and that VICs are known to exhibit perivascular characteristics in our model, it is plausible that NOX‐generated ROS might alter VIC function in a disease‐specific manner. The pathogenesis of CAVS involves a complex interplay between inflammation, endothelial dysfunction and VIC‐driven matrix remodelling [40]. NOX enzymes, particularly NOX2 and NOX4, are key sources of ROS in vascular and valvular cells. Increased NOX activity can promote oxidative stress, leading to the activation of pro‐fibrotic pathways such as TGF‐β/SMAD signalling. In calcified aortic valves, heightened oxidative stress is thought to contribute to the progression of disease by accelerating calcification [41, 42]. In the context of our findings, C‐VICs exhibited a significantly reduced ability to differentiate into perivascular cells and support vascularisation in vivo, as evidenced by the lower microvessel density in Matrigel plug assays. If oxidative stress levels differ between C‐VICs and NC‐VICs, this could explain, at least in part, their differential angiogenic potential. Increased ROS production in calcified regions might impair VIC function, either by directly affecting their differentiation capacity or through alterations in endothelial cell‐VIC interactions. The study by Hagler et al. [43] highlights how oxidative stress, specifically through NOX activation, can amplify TGF‐β signalling and contribute to fibrosis and extracellular matrix remodelling in valvular disease. Future studies should aim to quantify NOX activity and oxidative stress markers in C‐VICs and NC‐VICs to determine whether there is a correlation between oxidative stress levels and perivascular differentiation capacity.

Our study has several limitations. First, separation of different areas of valves was performed according to the macroscopic appearance of the valve and we have only distinguished between calcified and noncalcified areas. A more precise separation is possible between nondiseased, fibrotic and calcified areas [13]. However, our approaches separate completely pathologic calcified tissue and quite healthy or in the process of calcification tissue. This allows us to focus on functional properties of VICs involved in the calcification process that may be repressed when calcification is completed. The second limitation involves the potential underrepresentation or overrepresentation of specific VIC subpopulations during in vitro culture. Another significant challenge in VIC isolation is the potential contamination by other cell types, particularly vascular endothelial cells (VECs). Such contamination could compromise results, as studies have shown that both human and porcine VECs may undergo endothelial‐to‐mesenchymal transition [35, 44, 45]. In consequence, to improve the purity of our cultures, we added a CD31‐negative selection in our isolation procedure. Absence of E‐selectin in conditioned media confirms the purity of our VICs populations. We also used ECFCs, which are considered as a reference of vasculogenic cells in humans [20] to assess the perivascular potential of VICs. The use of these cells has enabled us to demonstrate the angiogenic potential of VICs. However, confirmation of the data obtained with VECs will be necessary in the future. Last, all experiments were performed in normoxia. Recent data underline the importance of hypoxia in the angiogenic potential of VICs, especially concerning proliferation capacities and secretion of angiogenic mediators [46].

All in all, this study illuminates the diverse properties of VICs in CAVS, driven by tissue alterations and VIC plasticity, particularly in perivascular differentiation. The functionally distinct cell subpopulations identified here will be pivotal in unravelling the molecular and cellular mechanisms underlying aortic valve homeostasis, shedding light on the intricate dynamics of calcification in CAVS. This research paves the way for innovative approaches to combat aortic valve disease at its roots.

## Author Contributions

**Adeline Blandinières:** conceptualization (equal), formal analysis (lead), writing – original draft (equal), writing – review and editing (equal). **Elisa Rossi:** data curation (equal), formal analysis (equal), investigation (equal), writing – review and editing (equal). **Nicolas Gendron:** data curation (equal), formal analysis (equal), investigation (equal), resources (equal), validation (equal), writing – review and editing (equal). **Jeanne Rancic:** data curation (equal), formal analysis (equal), investigation (equal), methodology (equal), resources (equal), writing – review and editing (equal). **Mickael Rosa:** data curation (equal), formal analysis (equal), writing – review and editing (equal). **Annabelle Dupont:** funding acquisition (equal), methodology (equal), project administration (equal), writing – review and editing (equal). **Salim Idelcadi:** formal analysis (equal), investigation (equal), writing – review and editing (equal). **Aurélien Philippe:** formal analysis (equal), investigation (equal), methodology (equal), resources (equal), writing – review and editing (equal). **Bastien Poitier:** formal analysis (equal), resources (equal). **Ivan Bièche:** formal analysis (equal), methodology (equal), writing – review and editing (equal). **Sophie Vacher:** formal analysis (equal), resources (equal). **Bernard Cholley:** conceptualization (equal), supervision (equal), writing – review and editing (equal). **Pascale Gaussem:** conceptualization (equal), supervision (equal), writing – review and editing (equal). **Sophie Susen:** conceptualization (equal), funding acquisition (equal), investigation (equal), methodology (equal), project administration (equal), writing – review and editing (equal). **David M. Smadja:** conceptualization (lead), data curation (supporting), formal analysis (equal), funding acquisition (equal), investigation (lead), methodology (lead), project administration (lead), resources (equal), software (supporting), supervision (lead), validation (lead), visualization (equal), writing – original draft (equal), writing – review and editing (equal).

## Conflicts of Interest

The authors declare no conflicts of interest.

## Supporting information


Table S1.

## Data Availability

All data and material information's are available on request from the authors.
