# Supplementary material for: Unveiling the Angiogenic Potential and Functional Decline of Valve Interstitial Cells During Calcific Aortic Valve Stenosis Progression
Source: J Cell Mol Med. 2025 Mar 30;29(7):e70511. doi: 10.1111/jcmm.70511 (PMC11955408; doi:10.1111/jcmm.70511)
Supplement: Supplementary file 1 — Table S1. [file JCMM-29-e70511-s001.docx]

**Supplementary data to Blandinieres et al: “Unveiling the Angiogenic Potential and Functional Decline of Valve Interstitial Cells During Calcific Aortic Valve Stenosis Progression”**

**Supplementary table 1: Primer sequences used**

| **Gene symbol** | **Primer sequence 5’-3’ U** | **Primer sequence 5’-3’ L** |
| --- | --- | --- |
| **Col1A1** | CCTCCGGCTCCTGCTCCTCTT | GGCAGTTCTTGGTCTCGTCACA |
| **Fn1** | AGTTGTCACCACTCTGGAGAATGT | GAAGCCAGTGATCGTCTCAGTCTT |
| **THY1** | GAACGTCACAGTGCTCAGAGACAAAC | TGTTCTGAGCCAGCAGGCTGA |
| **CD44** | CTTTCAATAGCACCTTGCCCAC | CCCTTCTATGAACCCATACCTGC |
| **CDH2** | GAGGGATCAAAGCCTGGAACAT | CGATTCTGTACCTCAACATCCCAT |
| **S100A4** | CTCGGGCAAAGAGGGTGACAA | GCTTCATCTGTCCTTTTCCCCAA |
| **TWIST1** | CGCCCCGCTCTTCTCCTCT | TGGACACGTCCTGCATCATCTCT |
| **VIM** | TCAGACAGGATGTTGACAATGCGT | CTGCAGCTCCTGGATTTCCTCTT |
| **NT5E** | AGCAGCATTCCTGAAGATCCAAG | TGAGAGGAGCCATCCAGATAGACA |
| **ENTPD1** | ACTTCCAGGGTGCCAGGATCAT | TGCTGAACCACCTTGTTTTCTGACT |
| **ADORA2B** | CCGTGGCAGTCGACAGATACCT | CCGTGACCAAACTTTTATACCTGAGC |
| **P2RY2** | CGTGGCGCTCTACATCTTCTTG | CAGACACAGCCAGGTGGAACAT |
| **P2RY12** | CTCTGCGCCTGGTAACACCAGT | CAGTGTAGAGCAGTGGGAAGAGGAC |
| **S1PR2** | CGCTCAAGCCACGCTGACA | CGATAAAGACGCCTAGCACGATG |
